# Gold Nanorod-Coated Hydrogel Brush Valves in Macroporous Silicon Membranes for NIR-Driven Localized Chemical Modulation

**DOI:** 10.3390/gels11010025

**Published:** 2025-01-01

**Authors:** Nafis Mustakim, Youngsik Song, Sang-Woo Seo

**Affiliations:** 1 Department of Electrical Engineering, The City College of New York, 160 Convent Avenue, New York, NY 10031, USA; nmustak000@citymail.cuny.edu (N.M.); ysong@ccny.cuny.edu (Y.S.); 2 Department of Engineering Technology, SUNY Westchester Community College, 75 Grasslands Rd., Valhalla, NY 10595, USA

**Keywords:** gold nanorod, NIR-actuation, PNIPAM valve, LBL deposition, chemical stimulation

## Abstract

A two-dimensional array of microfluidic ports with remote-controlled valve actuation is of great interest for applications involving localized chemical stimulation. Herein, a macroporous silicon-based platform where each pore contains an independently controllable valve made from poly(N-isopropylacrylamide) (PNIPAM) brushes is proposed. These valves are coated with silica-encapsulated gold nanorods (GNRs) for NIR-actuated switching capability. The layer-by-layer (LBL) electrostatic deposition technique was used to attach the GNRs to the PNIPAM brushes. The deposition of GNRs was confirmed by dark-field optical microscopy, and the localized surface plasmon resonance (LSPR) of the deposited GNRs was analyzed using UV-Vis spectra. To evaluate the chemical release behaviors, fluorescein dye was employed as a model substance. The chemical release properties, like OFF-state diffusion through the valve, the ratio between ON-state and OFF-state chemical release, and the rapidness of chemical modulation of the valve, were investigated, varying the PNIPAM brush thickness. The results indicate that enhancing the thickness of the PNIPAM brush in our platform improves control over the chemical modulation properties. However, excessive increases in brush length may lead to entanglement, which negatively impacts the chemical modulation efficiency.

## 1. Introduction

In synaptic interfaces of neurons, the receptors on the neuron cell membrane are modulated by controlling neurotransmitter concentrations, known as chemical stimulation [1]. It is considered one of the most promising strategies for neuromodulation due to its ability to mimic natural processes [2,3,4]. Unlike traditional electrical stimulation, which indiscriminately activates all neurons near the stimulation site and suffers from the current spread that limits spatial resolution, chemical stimulation offers unparalleled precision in targeting specific neural pathways or cell types [5,6]. Several studies have demonstrated the proof of concept using bulky structures to stimulate neurons chemically with ex-vivo setups [7,8,9]. However, these approaches rely on external pneumatic pumps or electro-osmosis to deliver small quantities of neurotransmitters, requiring complex control systems. To advance chemical stimulation platforms, particularly for retinal prostheses, significant miniaturization is essential. A densely packed, two-dimensional array of fluidic ports with individual chemical modulation capabilities is crucial for achieving high spatial resolution, presenting engineering challenges that demand microfabrication technology-based solutions [1,10].

Thermoresponsive polymers, especially poly(N-isopropylacrylamide) (PNIPAM), have recently gained great attention from the scientific community due to their ability to undergo temperature-actuated sol–gel phase transitions [11,12]. This unique temperature-responsive behavior has proven particularly beneficial for applications requiring localized chemical release, including drug delivery systems and controlled release of chemical payloads [13,14,15]. The tunability of its lower critical solution temperature (LCST) and easy integration with plasmonic nanoparticles have further amplified their utility in such biomedical applications. To leverage these properties, PNIPAM hydrogels have been integrated with various supporting structures to form temperature-actuated valves for various drug delivery applications. Surface-initiated atom transfer radical polymerization (SI-ATRP) of PNIPAM hydrogel provides unique advantages when the hydrogel is integrated with other structural platforms. Hydrogel chains can be directly grafted from a platform surface in a controlled manner. For example, PNIPAM hydrogel has been incorporated into nanoscale porous silicon film structures using SI-ATRP for hydrogel-based actuators and drug delivery platforms [16,17,18]. Composite membranes combining PNIPAM with polyethylene terephthalate track-etched porous films have shown potential for filtration applications [19]. However, the typical thickness of grafted hydrogels via the SI-ATRP process is limited to the nanometer scale, which restricts their use in drug delivery systems required with nanoscale-pore actuation. To utilize PNIPAM hydrogel in retinal chemical actuation, localized chemical release from UV-polymerized hydrogel/gold nanorod (GNR) composite embedded in a macroporous silicon membrane was demonstrated [20,21,22]. The macroporous silicon serves as a unique membrane platform with a predefined high density of pores, functioning as micron-scale fluidic channels, making it ideal for arrayed chemical stimulators. Combining surface-grafted PNIPAM brushes with the macroporous silicon membrane could offer a superior alternative due to their seamless integration and guided growth directly from the pore surface.

In this paper, the fabrication of two-dimensionally arranged PNIPAM brush-based valves on a macroporous silicon membrane by the SI-ATRP approach is reported. To grow the micron-scale PNIPAM brush, zerovalent copper (Cu^0^-coated plate) was used as an active catalyst. This approach improves the oxygen tolerance of the grafting process, which is one of the primary bottlenecks of polymer growth [23,24]. Moreover, silica-encapsulated GNRs were coated on the PNIPAM brushes using an electrostatic charged-based layer-by-layer (LBL) deposition technique. The GNR coating facilitates the PNIPAM brushes to be remotely actuated with near-infrared (NIR) light, which is relatively transparent in biological materials. As a result, it eliminates the need for complex and bulky external pumping systems to deliver small quantities of chemicals to the targeted stimulation sites. Additionally, the localized chemical modulation capabilities of the valves varying the PNIPAM brush thickness were investigated. Such a platform could be well-suited for the practical application of localized chemical stimulation of neurons.

## 2. Result and Discussion

### 2.1. Thermal Response of PNIPAM Brush-Based Valve

**Figure 1:**
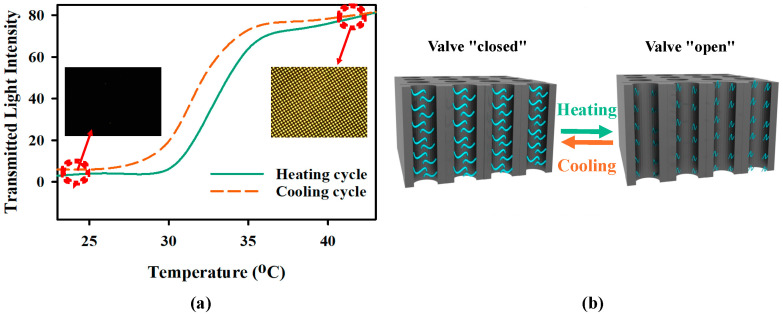
(**a**) Transmitted light through the pores of macroporous silicon-containing PNIPAM brushes as a function of temperature. In the insert, an optical microscope image of the transmitted light around 24 °C and 41 °C is shown. (**b**) Schematic diagram of the temperature-dependent valve operation.

The thermoresponsive behavior of the valve was investigated by observing light transmission through the PNIPAM brush-grown pores of the macroporous silicon membrane at varying temperatures. The membrane sample was placed in a custom-made aluminum chamber and filled up with water. The chamber had two openings sealed with cover glass slides to let the light pass through the sample. The block was placed under an upright microscope and illuminated with white light. Using a heating block, the temperature of the sample holder was varied between 23 °C and 42 °C. Figure 1a shows the transmitted light intensity through the pore for varying temperatures, recorded with a microscope camera. The transmitted light through the membrane’s pores was minimal at a temperature of 23 °C. The PNIPAM brush remained swollen at this temperature, and the elongated brush closed the pore opening, causing minimal light to transmit. So, the valves were in a “closed” state, as shown in Figure 1b. The brush started collapsing as the temperature increased. As a result, the pores started to open up and the opening increased as the temperature increased, causing the transmitted light intensity to rise. The sharp transition of the transmitted light was observed from 30 to 35 °C due to the phase transition behavior of PNIPAM. The valves were in a fully “open” state, as shown in Figure 1b, when the temperature increased beyond 35 °C. As a result, the intensity of transmitted saturated for the temperature beyond ~35 °C. When the temperature was brought down from 42 °C to 23 °C, the intensity of the transmitted light returned to its initial condition as the valves “closed”. Hysteresis can be observed during the phase transition, which was also reported in previous studies [25].

**Figure 2:**
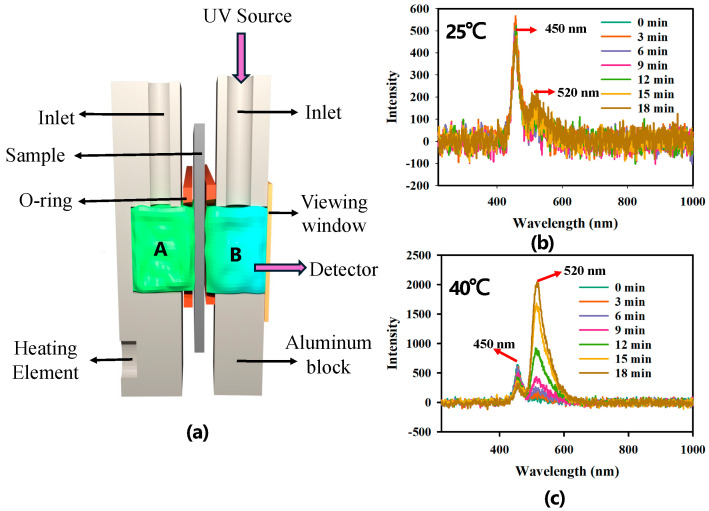
(**a**) Schematic diagram of the experimental setup for measuring UV-Vis response of dye transfer from chamber A to chamber B. (**b**) The UV-Vis spectra of diffused fluorescent dye in chamber B over 18 min at 25 °C. (**c**) The UV-Vis spectra of diffused fluorescent dye in chamber B over 18 min at 40 °C.

Next, the effectiveness of the valve in controlling the flow of chemicals between two chambers was investigated. Fluorescein dye was used as the model chemical, and the UV-Vis spectrum was used to quantify it. Figure 2a shows the schematic of the experimental design. The membrane sample with the valves was placed between two aluminum blocks containing chambers A and B.

Chamber A was used as the reservoir, and the amount of chemical substance transported to chamber B was observed. O-rings were used to prevent leakage from the chambers. After mounting the sample in the setup, chambers A and B were each filled with 500 μL of water. Then, 40 μL of dye (molar concentration: 1 M; 0.376 g fluorescein sodium salt in 1 mL of DI water) was added to chamber A. The same amount of DI water was added to chamber B to balance the liquid content in both chambers. A fiber optic cable with the UV source was plugged into chamber B, and a detector was mounted with the viewing window to analyze the transported dye from chamber A to B with UV-Vis spectrum. Figure 2b shows the UV-Vis response of the chamber B at room temperature (23 °C) over 18 min. The peak ~450 nm wavelength corresponds to the UV source, and ~515 nm is due to the emission of the fluorescent dye. The valves were closed at room temperature. So, the UV-Vis spectrum did not show dramatic variation for 18 min. On the other hand, the fluorescent emission intensity peak (~515 nm) increased over time at 40 °C because the valves were open at that temperature, allowing the dye from chamber A to diffuse through the membrane to chamber B. This increased amount of dye molecules in chamber B absorbed even more UV light, which caused the UV absorption peak (~450 nm) to decrease with time.

### 2.2. Effect of PNIPAM Brush Length on Valve Operation

Using the experimental setup described in Figure 2a, the effect of PNIPAM polymer brush length on the valve operation was analyzed. Four samples were prepared with varying brush lengths by controlling the polymerization time, as the polymerization time during the SI-ATRP process determines the polymer brush thickness [18]. The chosen polymerization times were 30 min, 60 min, 90 min, and 120 min. This implies that the brush length was the shortest when the polymerization time was chosen to be 30 min. On the other hand, the longest brush length was obtained for the polymerization time of 120 min.

**Figure 3:**
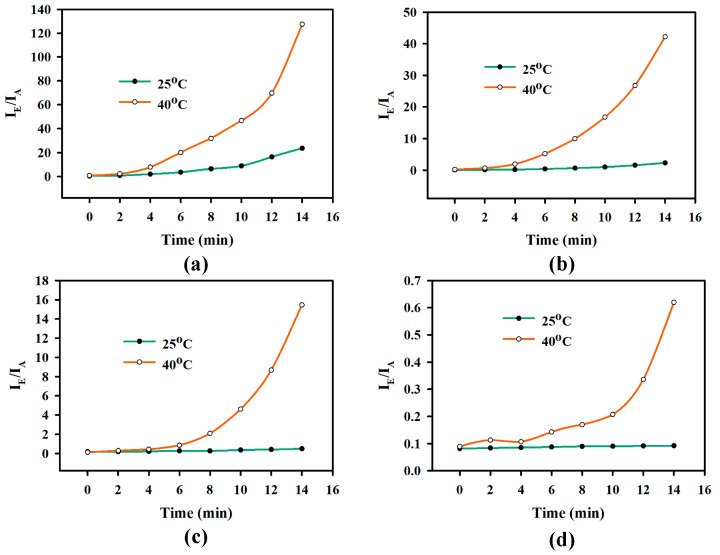
The ratio of the emission and absorption intensity of the fluorescent dye of chamber B at 25 °C and 40 °C for macroporous silicon containing PNIPAM brushes polymerized for (**a**) 30 min, (**b**) 60 min, (**c**) 90 min, and (**d**) 120 min.

Figure 3 shows the ratio of the emission intensity of the dye and the absorption intensity of the UV source as a function of time at temperatures 25 °C and 40 °C. The plot in Figure 3 was derived from similar experiments, like Figure 2b,c, carried out for samples with different PNIPAM brush lengths. The ratio helps to eliminate side effects from the fluctuation of UV light intensity. The ratio of the emission intensity and absorption intensity (I_E_/I_A_) of the fluorescein dye at chamber B can be defined as follows:

$$\frac{I_{E}}{I_{A}}=\frac{Emission\;peak\;intensity\;at\;520\;nm}{Absorption\;peak\;intensity\;at\;450\;nm\;}$$

As the valves are in the “closed” or “OFF” state at 25 °C, diffusion through the membrane is expected to be minimal. As a result, the ratio of the emission and absorption intensity is expected to remain constant. On the other hand, at 40 °C, the dye concentration is expected to increase in chamber B at 40 °C since the valves are in the “open” or “ON” state. So, the ratio of the emission and absorption intensity is expected to increase. Figure 3a is the plot for 30 min, Figure 3b is for 60 min, Figure 3c is for 90 min, and Figure 3d is for 120 min of polymerization time. In all cases, the ratio of emission and absorption intensity was minimal when valves were OFF and increased dramatically over time when the valves were ON. At 25 °C, the ratio of the emission and absorption intensity increased steadily over 14 min for the 30 min polymerized sample, as shown in Figure 3a. However, this increase was observed to be (Figure 3b) less prominent for the 60 min polymerized sample. Moreover, the ratio of emission and absorption intensity, as shown in Figure 3c,d, was relatively constant for 14 min for the 90 and 12 min polymerized samples. This result implies that the PNIPAM brush length needs to be long enough to close up the macroporous membrane’s pore opening to stop the diffusion of chemical molecules when the valve is in the OFF state. From the result in Figure 3, we can see that the PNIPAM brush was not long enough for 30 min of polymerization, so the diffusion of dye through the valve was observed during the OFF state. As the polymerized time was increased, the brush length became long enough to allow minimum diffusion of dye during the OFF state. On the other hand, at 40 °C, the ratio of the emission and absorption intensity showed the highest increase in the sample with 30 min polymerization time, and it gradually decreased with the increase in polymerization time. In the ON state, the pore opening was larger for the valve with the smaller PNIPAM brushes compared to the valve with longer PNIPAM brushes. However, the control of the chemical release between ON and OFF states is dependent on the difference in the amount of chemicals released in the two states. The difference in the final value (after 14th min) of the ratio of the emission and absorption intensity of the ON and OFF states was compared to estimate the efficiency of the valve. The ratio of the emission and absorption intensity at 25 °C was 5.52 times, 20 times, and 31 times the value at 40 °C for Figure 3a,b,c, respectively. The contrast between the chemical actuation of ON and OFF states became more rapid, along with minimizing diffusion during the OFF state. However, the ratio of emission and absorption intensity at 25 °C was 6.72 times the value at 40 °C for Figure 3d. Brush length beyond some length might cause it to entangle with other brushes grown from the side walls of the porous silicon. Further investigation is needed to understand the threshold length that causes the PNIPAM brush to entangle with each other.

### 2.3. Photothermal Response of the PNIPAM Brush-Based Valve

**Figure 4:**
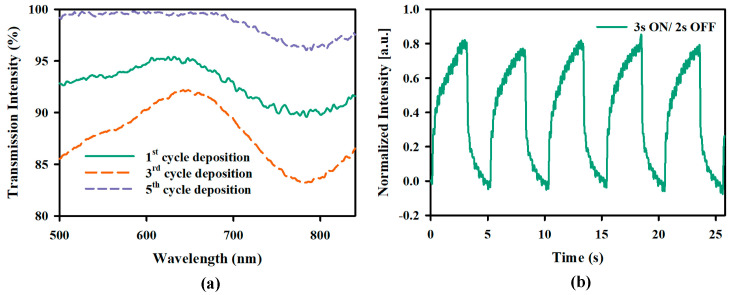
(**a**) UV-Vis spectra after the 1st, 3rd, and 5th cycle of GNR deposition on a glass sample functionalized with PNIPAM film by SI-ATRP process. (**b**) Normalized transmitted light intensity variation through the pores of a macroporous silicon substrate containing PMIPAM brushes with 3 s ON 2 s OFF NIR pulse (60 mW/mm^2^).

The UV-Vis transmission spectrum of the deposited GNR was observed by depositing GNR on a PNIPAM brush-coated glass substrate to ensure that the LSPR peak remained in the NIR region after multilayer deposition. Figure 4a shows the normalized transmission intensity as a function of wavelength. The plots show a transmission dip around 790 nm due to the longitudinal mode of LSPR. The light around these wavelengths is absorbed by the GNR and converted to heat. Each cycle of LBL deposition increased the number of GNR deposited on the sample, enhancing the transmission dip. To observe the NIR actuated valve operation, the sample was placed in the aluminum block described for the experiment in Figure 1a. However, laser pulses (60 mW/mm^2^) with 3 s ON and 2 s OFF time were illuminated over the sample instead of changing the temperature with a heating element, and the corresponding transmission intensity change through the sample’s pores was observed. Figure 4b shows the response of the normalized intensity under pulsed laser illumination. During the ON cycle, the normalized transmission through the pores increased due to the collapse of the PNIPAM brushes as the local temperature rose beyond LCST temperature. As a result, the valves were “opened”. On the other hand, the valves went back to the “closed” state when the laser was OFF due to the heat dissipation in the surrounding medium (water).

### 2.4. Localized Chemical Release from the PNIPAM Brush-Based Valve

**Figure 5:**
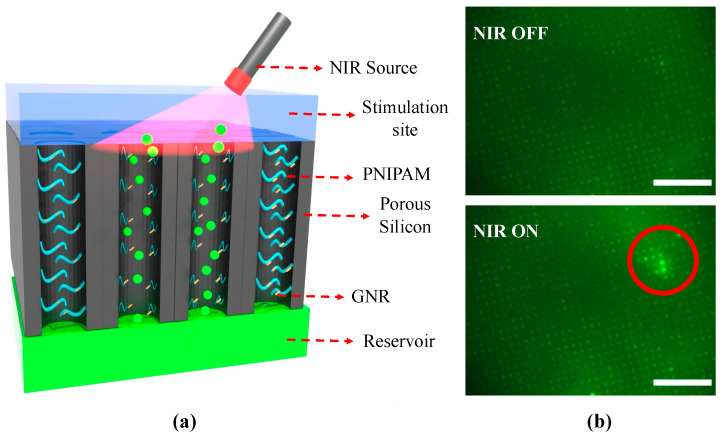
(**a**) Schematic diagram of the NIR-actuated localized chemical release from the proposed platform. (**b**) Optical microscope image of the fluorescent response of the stimulation site under UV illumination when NIR is ON and OFF (60 min polymerization). The NIR illuminated region is marked in red. The scale bar is 100 μm.

The localized chemical release property of the valve array was investigated using fluorescein dye as a model chemical in a flow control measurement. The experimental setup used for this study is discussed in detail in our previously reported study [22]. Briefly, the sample was placed in a custom-made 3D-printed model so that the backside of the membrane sample was connected to the dye reservoir and the topside was connected to the stimulation site containing pure water. The water was flushed at a constant rate to wipe out the released chemicals after each NIR ON cycle. This helps to quantify the released chemical from each pulse. NIR light was focused on the micron-scale area of the sample using a microscope. The topside of the sample was illuminated with a UV source, and the corresponding fluorescent signal was captured with the same microscope equipped with dichroic UV and NIR filters. Figure 5a shows the schematic diagram of the NIR light-actuated localized chemical release behavior of the proposed device. When the NIR light was ON, the valve opened, allowing the dye to transport from the reservoir side to the stimulation site through the porous silicon channel. However, when the NIR light was OFF, the valve closed, and the dye transportation through the porous silicon channel halted.

Figure 5b shows the optical microscope image of the fluorescent response during the ON/OFF operation of the NIR. The fluorescent dye intensity in the NIR-actuated region (marked in red) was higher compared to the dye intensity when NIR was OFF, signifying the localized dye release at the NIR-actuated area. Supplementary Video S1 shows the dynamic response of the NIR-actuated dye modulation of the proposed platform.

**Figure 6:**
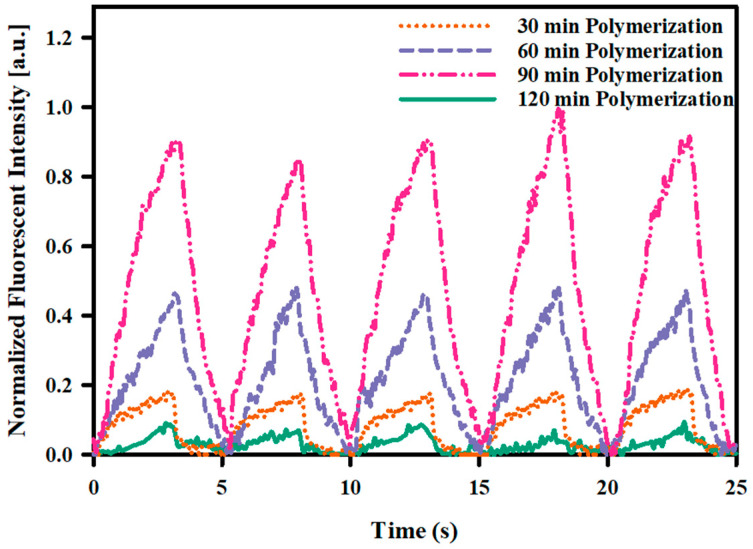
Normalized fluorescent intensity variation in laser-actuated area of the stimulation site with a 3 s ON 2 s OFF laser pulse for samples with varying PNIPAM brush length due to different polymerization times.

Figure 6 shows the difference in modulation depths (difference of diffused dye between the ON and OFF state of the valve) of actuated dye through the valves with varying PNIPAM brush thickness for a 3 s ON- 2 s OFF NIR pulse of 60 mW/mm^2^. During the NIR ON cycle, the fluorescent intensity increased because the valves were opened in the actuated area, allowing the dye to be released. The fluorescent intensity decreased to the initial state during the NIR OFF cycle because the valves closed. It was found that the PNIPAM brush thickness played a crucial role in the actuation property of the valves. As discussed in Figure 3, four samples were prepared, varying the polymerization time to prepare valves with variable PNIPAM brush thickness. Observing 30, 60, and 90 min polymerized samples from Figure 6, it can be stated that the longer PNIPAM brush thickness yields more rapid actuation of chemicals. The difference in chemical flow through the macroporous channel was much more significant if the length of the PNIPAM brush thickness increased.

However, for the sample containing the valves with a polymerized time of 120 min, the fluorescent intensity increase during the NIR ON cycle was even lower than the 30 min polymerized sample. The reason for such a low chemical actuation might be the same as explained in Figure 3d, which is the entangled PNIPAM brush cannot properly shrink to make enough space within the channel for rapid dye molecule movement. Thus, the PNIPAM brush thickness plays a key role in the chemical actuation performance of the proposed platform. The result shows excellent agreement with the experiment carried out in Figure 3, signifying that the behaviors of the valves remain the same for localized release. The localized release platform can be designed to be implanted in either the epiretinal or sub-retinal region, with a connection to a larger external reservoir [10]. This external reservoir could potentially be placed in a location such as behind the ear. While the lifespan of the platform has not yet been determined, the use of relatively biocompatible and durable materials in the proposed platform suggests that its lifespan can meet the typical standards for implantable devices through its controlled operation and additional protective coatings [26,27,28,29]. In future work, the efficacy of localized chemical release must be evaluated on biological cells by carefully designing in vitro experiments. One potential direction for future experiments is to culture retinal cells on the proposed platform and observe their calcium transient responses when localized chemical stimulation is applied using neurotransmitters released in arrayed valves by light.

## 3. Conclusions

A localized chemical stimulation platform has significant potential as an artificial synaptic interface, allowing for the replacement of degenerated neurons, which do not release neurotransmitters. The primary challenges facing existing chemical stimulators are miniaturization and simplified operation without complex chemical pumping systems. In this study, a GNR-coated PNIPAM brush-based valve array on a macroporous membrane for NIR-actuated localized chemical release is reported. The PNIPAM brushes were fabricated on a macroporous silicon membrane, using a modified SI-ATRP process, and its thermo-responsive behavior was observed. The PNIPAM brush thickness was controlled by varying polymerization times. The brush thickness was around 0.65 µm, 0.75 µm, 1.2 µm, and 1.5 µm for polymerization times of 30 min, 60 min, 90 min, and 1080 min, respectively. GNR was coated on the PNIPAM brushes by the LBL deposition process to have NIR-induced temperature control. The charge of the GNR was modified using silica, PDDA, and PSS, and the modification was confirmed with the gel electrophoresis technique. Dark-field microscope images of the sequential deposition of GNR were recorded to observe the deposition. The UV-Vis spectrum of the deposited GNR was also measured to investigate if the LSPR peak remained after deposition. The fluorescein dye transport through this valve during OFF and ON states was measured as a function of time. During the OFF state, the fluorescent intensity increase was minimal compared to the ON state. The brush length of the PNIPAM played an important role in creating the contrast between the fluorescent intensity during ON and OFF states. The ON state fluorescent intensity was 5.52 times, 20 times, and 31 times compared to the OFF state for 30 min, 60 min, and 90 min, respectively. However, this contrast of fluorescent intensity between ON and OFF states decreased to 6.72 times when the polymerization time of the PNIPAM brush was 120 min. The NIR-actuated localized valve operation was observed by analyzing the light transmission through the porous membrane. The chemical modulation behavior of the valve was investigated using fluorescein dye as a model drug, as well as the effect of the PNIPAM brush length on the valve performance.

## 4. Materials and Methods

N-isopropylacrylamide (NIPAAm), 2-bromoisobutyryl bromide (BIBB, 98%), aminopropyl triethoxysilane (APTES, 98%), N,N,N′,N″,N″-pentamethyldiethylenetriamine (PMDETA), and silver nitrate (AgNO_3_) were purchased from TCI, Portland, OR, USA. Chloroauric acid (HAuCl_4_), sodium borohydrate (NaBH_4_), cetyltrimethylammonium bromide (CTAB), poly(styrene sulfonate sodium) (PSS), and poly(diallyldimethylammonium chloride) (PDDA) were purchased from Sigma Aldrich, St. Louis, MO, USA. Ascorbic acid (C_6_H_8_O_6_) and triethylamine (TEA) were purchased from VWR, Radnor, PA, USA. Agarose gel was purchased from IBI scientific, Dubuque, IA, USA. All chemicals were used as received without further purification. Deionized (DI) water (Reagent Grade, Electron Microscopy Sciences, Inc., Hatfield, PA, USA) was used for all aqueous solutions.

### 4.1. GNR Preparation and Charge Modification

GNR was prepared using a well-established seed-mediated method [30]. The excess CTAB in GNR suspension was removed by centrifuging twice with fresh DI water. Next, the GNR was coated with silica to preserve the absorption peak after multilayer deposition. The silica coating process is discussed in our previous work [31]. Following the silica coating, the surface charge of the GNR was modified using polyelectrolyte coating. Silica-coated GNR is inherently negatively charged [32]. With polyelectrolyte coating, the surface charge of the silica-encapsulated GNR was modified to both positive and negative. The silica-coated GNR was suspended in 0.2 wt.% solution of PDDA in 1 mM NaCl and stirred overnight to alter the surface charge to positive. The PDDA-modified silica-coated GNR (PDDA@Si-GNR) was centrifuged and stirred overnight in 0.2 wt.% PSS in 1 mM NaCl solution to obtain negatively charged PSS@Si-GNR. Following the polyelectrolyte coating, the charge-modified silica-coated GNR was centrifuged and resuspended in 1 mM NaCl solution.

**Figure 7:**
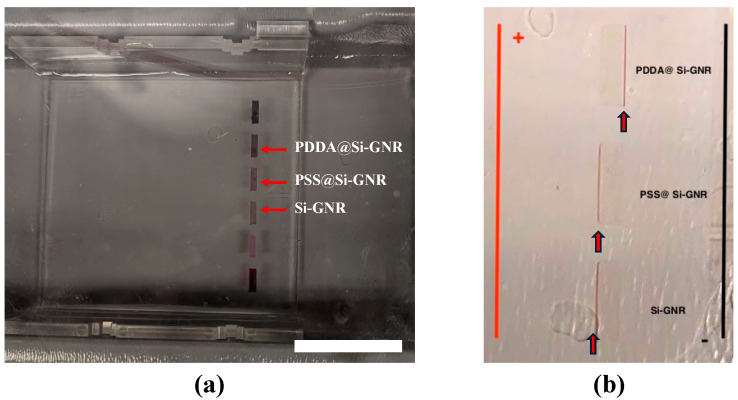
Identification of surface charge of gold nanorod using gel electrophoresis. Photograph of the nanorod suspension in gel electrophoresis setup (**a**) before applying DC bias and (**b**) after applying DC bias. The scale bar is 2.50 cm.

The gel electrophoresis technique was utilized to identify the surface charge of the GNR. Several previous works have shown the possibility of mobilizing or separating GNR under the influence of a DC electric field based on their charge and shape [33,34,35]. In our procedure, the loading buffer was made of a 1:1 (*v*/*v*) ratio of glycerol and water. Next, 30 μL of nanorod suspension was mixed with 5 μL loading buffer. Then, the suspension with the loading buffer was pipetted into the well of 0.5 wt.% agarose gel. Then, a 95 V DC bias was applied to the electrodes with a power supply. The GNR started to migrate in the direction of the electrode with opposite charges within a few minutes after the bias was applied. Figure 7a shows the photograph of the gel electrophoresis setup before applying the bias. Figure 7b shows the close-up snap of the well after application of the DC bias. A band of the PDDA-modified silica-coated GNR formed near the wall of the well closer to the negative electrode due to its positive charge. Similarly, PSS-modified silica-coated GNR and silica-coated GNR formed a band near the wall close to the positive electrode as a result of their negative charge.

The migration of the nanorod band halted at the edge of the wall of the well of agarose gel when a DC bias of 95 V was applied. However, the migration of GNR inside the agarose gel was observed when agarose gel containing 0.2 wt.% or less gel was made. Handling became challenging for such low concentrations as the gels became extremely soft and fragile. As identifying the surface charge was the main objective of this study, getting the band of the nanorod along the wall of the well was satisfactory enough. Further research might be needed to quantitatively compare the surface charge of different samples through gel electrophoresis.

### 4.2. Fabrication of PNIPAM Brush-Based Valve

Macroporous silicon membranes were chosen as the backbone of the valve array because of their excellent structural tunability, nontoxicity, and versatility [36,37]. The backside of the one-side opened porous silicon substrates (Smart Membrane, Halle (Saale), Germany) were lapped and polished with alumina slurry to make the silicon membrane with the through-hole array. The membrane was oxidized a few times, followed by buffered oxide etching to ensure a pore diameter of ~5 μm. The details of the macroporous silicon membrane preparation are discussed in our previous work [21]. All the macroporous silicon membrane samples presented in this study were processed concurrently to ensure similar experimental conditions.

The macroporous silicon membrane was sonicated in acetone for 1 min and thoroughly cleaned with acetone, methanol, and isopropyl alcohol. After solvent cleaning, the cleaned macroporous silicon membrane was immersed in piranha solution (3:1 *v/v* of H_2_SO_4_:H_2_O_2_) to terminate the surface of pore walls with hydroxyl group (–OH) for 30 min at room temperature. After thorough rinsing with DI water, the sample was immersed in 2 vol.% APTES in degassed toluene for amino termination (–NH_2_) for 30 min. During the ATPES functionalization process, the macroporous silicon membrane was briefly sonicated several times to remove physiosorbed amino groups on the surface. After amino functionalization, the macroporous silicon membrane was cleaned with toluene and ethanol. Next, the sample was baked at 110 °C for 30 min in a convection oven. The sample was then immersed in 1 vol.% TEA in degassed toluene while stirring by a magnet stirrer, and 1 vol.% BIBB was added drop wisely to the solution and kept for 2 h with stirring, followed by rinsing with toluene and ethanol. Next, the sample was placed on a custom-made PTFE holder, which maintains a gap of 800 nm on both sides of the macroporous silicon membrane and 200 nm Cu layer on glass slides. The 200 nm Cu film was deposited by the thermal evaporation method at the base pressure of 8 × 10^−6^ torr. NIPAM monomer solution was prepared by mixing 6 mL degassed DI water, 2 mg NIPAM, 2 mL MeOH, and 75 μL of PMDETA. The PTFE holder with macroporous silicon membrane sandwiched by two Cu-coated glass slides was placed inside a beaker in an N_2_-filled glove bag, and the prepared monomer solution was gently introduced by a pipette. Following the introduction of the monomer solution, the setup was left for polymerization in the N_2_ environment. The polymerization time varied depending on the length of the polymer brush. Four samples were prepared with polymerization times of 30 min, 60 min, 90 min, and 120 min. The thicknesses of the dried PNIPAM brush layer grown on a flat 100 nm silicon dioxide/silicon substrate with different polymerization times were measured using a thin-film thickness measurement system (Filmetrics 20, San Diego, CA, USA), as shown in Table 1 for reference. Spectral reflectance was recorded over a wavelength range from 400 nm to 1000 nm. Based on the complex matrix form of the Fresnel equations, the thickness of the PNIPAM brush layer was estimated by comparing the measured reflectance spectrum with the calculated one. The accuracy of this estimation was confirmed, achieving over 90% of a goodness of fit between the measured and calculated reflectance spectra.

### 4.3. GNR Coating of PNIPAM Brush-Based Valve

GNRs were coated on the PNIPAM brush following the layer-by-layer (LBL) electrostatic deposition technique. In this technique, the substrate and the nanorods are modified to opposing charges to establish electrostatic attraction between them [38]. After the PNIPAM brush growth, the sample was immersed in 0.2 wt.% PDDA in 1 mM NaCl solution for 30 min. To ensure complete wetting, the PDDA solution with the sample was desiccated and left undisturbed for 30 min. Next, the sample was dried with nitrogen blow and immersed in a PSS-modified silica-coated GNR solution (negatively charged). Degassing was performed to ensure the pores of the porous silicon were filled with the solution. The sample was kept in the solution for 30 min. This completed the 1st cycle deposition of GNR on the PNIPAM brushes. Next, the same sample was immersed in 0.2 wt.% PSS in 1 mM NaCl solution to make it negatively charged. Then, the sample was immersed in PDDA-modified silica-coated GNR solution for 30 min. This led to the 2nd cycle of GNR deposition. Then, the steps of the 1st deposition cycle were repeated, and so on. In this way, five deposition cycles were carried out.

**Figure 8:**
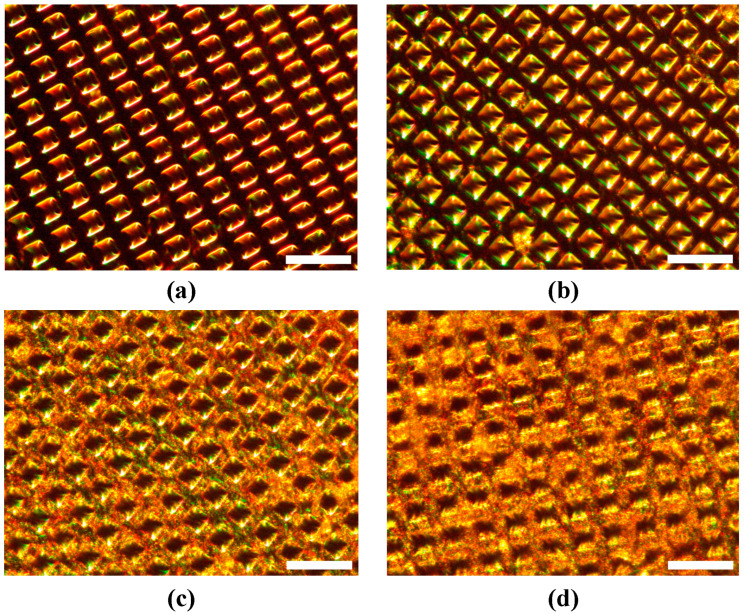
Dark-field microscope image of the macroporous silicon membrane with PNIPAM brushes after (**a**) 0th, (**b**) 1st, (**c**) 3rd, and (**d**) 5th cycle of GNR deposition. The scale bar is 20 μm.

Figure 8 shows the dark-field microscope image of the PNIPAM grafted macroporous silicon membrane at the 0th, 1st, 3rd, and 5th cycle of GNR deposition. Light scattering from the surface became more prominent with the increase in deposition cycles due to a higher amount of deposited GNR.

## Figures and Tables

**Table 1:** Dry PNIPAM brush length for different polymerization times.

Polymerization Time (min)	Dry PNIPAM Brush Length (μm)
30	0.65 ± 0.03
60	0.75 ± 0.05
90	1.2 ± 0.06
1080	1.5 ± 0.08

## Data Availability

All data are included in the manuscript.

## Supplementary Materials

The following supporting information can be downloaded at https://www.mdpi.com/article/10.3390/gels11010025/s1, Video S1: Localized fluorescent release through the microvalve when actuated with a 3s ON/2s OFF NIR pulse of 60 mW/mm^2^.
